# Theophylline–gentisic acid (1/1)

**DOI:** 10.1107/S1600536809031031

**Published:** 2009-08-08

**Authors:** Srinivasulu Aitipamula, Pui Shan Chow, Reginald B. H. Tan

**Affiliations:** a Institute of Chemical and Engineering Sciences, A*STAR (Agency for Science, Technology and Research), 1 Pesek Road, Jurong Island, 627833 Singapore; b Department of Chemical & Biomolecular Engineering, National University of Singapore, 4 Engineering Drive 4, 117576 Singapore

## Abstract

In the title 1:1 cocrystal, C_7_H_8_N_4_O_2_·C_7_H_6_O_4_, the anti-asthmatic drug theophylline (systematic name: 1,3-dimethyl-7*H*-purine-2,6-dione) and a non-steroidal anti-inflammatory drug, gentisic acid (systematic name: 2,5-dihydroxy­benzoic acid) crystallize together, forming two-dimensional hydrogen-bonded sheets involving N—H⋯O and O—H⋯N hydrogen bonds. The overall crystal packing features π–π stacking inter­actions [centroid–centroid distance = 3.348 (1) Å]. The cocrystal described herein belongs to the class of pharmaceutical cocrystals involving two active pharmaceutical ingredients which has been relatively unexplored to date.

## Related literature

For characterization of the title cocrystal by Fourier Transform Infrared Spectroscopy, see: Childs *et al.* (2007 ▶). For a detailed study on theophylline monohydrate see: Khankari & Grant (1995 ▶). For recent cocrystals of the theophylline, see: Trask *et al.* (2006 ▶); Lu *et al.* (2008 ▶). For recent cocrystals involving two or more active pharmaceutical ingredients, see: Aitipamula *et al.* (2009 ▶); Bhatt *et al.* (2009 ▶); Vishweshwar *et al.* (2005 ▶); Caira (2007 ▶); Childs (2007 ▶); Childs *et al.* (2007 ▶); Fleischman *et al.* (2003 ▶); Shan & Zaworotko (2008 ▶).
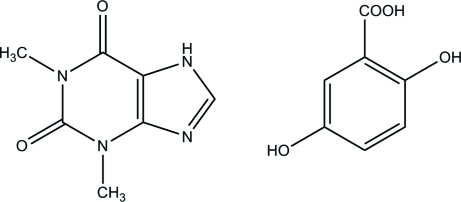


## Experimental

### 

#### Crystal data

C_7_H_8_N_4_O_2_·C_7_H_6_O_4_
*M* *_r_* = 334.29
Triclinic, 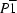

*a* = 7.0989 (14) Å
*b* = 8.0543 (16) Å
*c* = 13.034 (3) Å
α = 86.08 (3)°
β = 81.27 (3)°
γ = 74.14 (3)°
*V* = 708.3 (3) Å^3^
*Z* = 2
Mo *K*α radiation
μ = 0.13 mm^−1^
*T* = 110 K
0.24 × 0.22 × 0.13 mm

#### Data collection

Rigaku Saturn CCD area-deterctor diffractometer
Absorption correction: multi-scan (Blessing, 1995 ▶) *T*_min_ = 0.971, *T*_max_ = 0.984
10245 measured reflections
3478 independent reflections
3302 reflections with *I* > 2σ(*I*)
*R*_int_ = 0.021

#### Refinement

*R*[*F* ^2^ > 2σ(*F* ^2^)] = 0.046
*wR*(*F* ^2^) = 0.127
*S* = 1.08
3478 reflections
235 parameters
H atoms treated by a mixture of independent and constrained refinement
Δρ_max_ = 0.36 e Å^−3^
Δρ_min_ = −0.33 e Å^−3^

### 

Data collection: *CrystalClear* (Rigaku, 2008 ▶); cell refinement: *CrystalClear*; data reduction: *CrystalClear*; program(s) used to solve structure: *SHELXS97* (Sheldrick, 2008 ▶); program(s) used to refine structure: *SHELXL97* (Sheldrick, 2008 ▶); molecular graphics: *X-SEED* (Barbour, 2001 ▶); software used to prepare material for publication: *SHELXTL* (Sheldrick, 2008 ▶) and *PLATON* (Spek, 2009 ▶).

## Supplementary Material

Crystal structure: contains datablocks global, I. DOI: 10.1107/S1600536809031031/pb2002sup1.cif

Structure factors: contains datablocks I. DOI: 10.1107/S1600536809031031/pb2002Isup2.hkl

Additional supplementary materials:  crystallographic information; 3D view; checkCIF report

## Figures and Tables

**Table 1:** Hydrogen-bond geometry (Å, °)

*D*—H⋯*A*	*D*—H	H⋯*A*	*D*⋯*A*	*D*—H⋯*A*
N1—H1⋯O1^i^	0.95 (2)	1.85 (2)	2.8000 (16)	177.2 (17)
O6—H6⋯O2	0.92 (2)	1.83 (2)	2.7478 (14)	173.9 (18)
O5—H5⋯O3^ii^	0.92 (2)	2.24 (2)	2.8503 (16)	122.6 (17)
O5—H5⋯O3	0.92 (2)	1.87 (2)	2.6617 (15)	142.3 (19)
O4—H4*A*⋯N2^iii^	0.99 (3)	1.68 (3)	2.6596 (16)	171 (2)

Symmetry codes: (i) 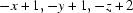; (ii) 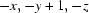; (iii) 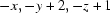.

**Fig. 1.**
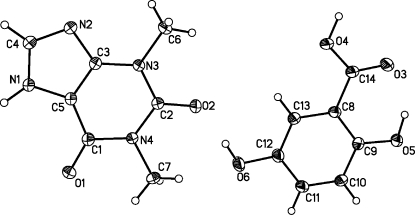
The molecular structures of theophylline and gentisic acid, with atom labels and 50% probability displacement ellipsoids for non-H atoms.

**Fig. 2.**
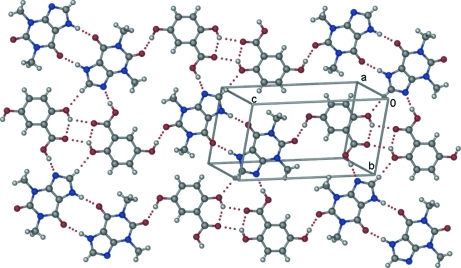
Part of the crystal structure of the title cocrystal, showing formation of a hydrogen bonded sheet in the (21–1) plane.

**Fig. 3.**
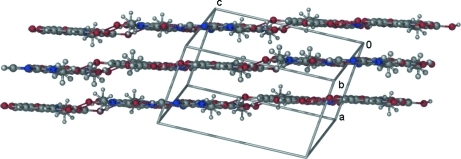
Part of the crystal structure of the title cocrystal, showing the π-π stacking interaction between two layers.

## supplementary crystallographic information

### Comment

Theophylline (1,3-dimethyl-7*H*-purine-2,6-dione) is a drug used in the treatment of respiratory diseases such as asthma. It has been reported that theophylline forms a monohydrate as a function of relative humidity and poses challenges in the formultion stages (Khankari and Grant, 1995). Using the cocrystallization as an aid to improve the physical stability, several theophylline cocrystals with dicarboxylic acids have been prepared and studied for their physical stability (Trask *et al.*, 2006, Childs *et al.*, 2007). Cocrystals which involve two or more active pharmaceutical ingredients (APIs) are relatively unexplored solid forms of APIs which have potential relevance in the context of combination drugs for pharmaceutical drug development (Aitipamula *et al.*, 2009, Bhatt *et al.*, 2009). We have recently reported trimorphs of a pharmaceutical cocrystal involving two APIs, namely ethenzamide (2-ethoxybenzamide), and gentisic acid and shown that the dissolution rate of the cocrystal polymorphs improved by two times when compared to the parent ethenzamide (Aitipamula *et al.*, 2009). In the present paper, we report a 1:1 cocrystal of theophylline with gentisic acid and analyzed the hydrogen bonding.

The crystal structure of the title cocrystal contains each one molecule of theophylline and gentisic acid in the asymmetric unit (Fig. 1). In the structure, two molecules of theophylline which are related by an inversion centre form a dimer involving N—H···O hydrogen bonds (Table 1). Hydroxy atom O5 of the gentisic acid acts as an intramolecular O—H···O hydrogen bond donor to the carbonyl of carboxyl group and also involves in a bifurcated O—H···O hydrogen bond to atom O3 at (-*x*, -*y* + 1, -*z*) (Fig. 2). Hydroxy atom O4 acts as a hydrogen bond donor to atom N2 of the theophylline at (-*x*, -*y* + 2, -*z* + 1), thus generating chains of alternating dimers of theophylline and gentisic acid running parallel to [21–1]. In addition, there is a C—H···O hydrogen bond between C4 of the theophylline and O5 of the gentisic acid. The 5-hydroxyl group (O6) of the gentisic acid acts as a hydrogen bond donor to atom O2 of the theophylline at (1 + *x*, -1 + *y*, 1 + *z*), thus generating a hydrogen bonded sheet parallel to the (21–1) plane (Fig. 2). The crystal structure is further stabilized by a π-π interaction involving pyrimidine ring of theophylline and phenyl ring of gentisic acid: *Cg1*···*Cg2* (*x, y, z*) = 3.348 (1) Å, where *Cg1 *and *Cg2* denote the centroids of N3/C2/N4/C1/C5/C3 of the theophylline and C8—C13 of the gentisic acid, respectively (Fig. 3).

Zaworotko and co-workers distinguished between two types of hydrogen bonding possibilities in cocrystal structures depending on whether the interacting complementary functional groups are the same or different (Fleischman *et al., *2003). In type I, an API forms hydrogen bonds like in pure structure, *e.g.* dimers, catemers, *etc*. (homosynthons) and such units are connected by cocrystal former spacer, and in type II, both the API and cocrystal former involve in heterosynthon formation. The title cocrystal belongs to type I, in which both the theophylline and gentisic acid molecules form dimers involving homosynthons, and such dimers are connected *via* O—H···O hydrogen bonds (Fig. 2).

### Experimental

Equimolar quantities of theophylline and gentisic acid (purchased from Aldrich) were dissolved in methanol upon heating. The solution was set aside to crystallize providing crystals that belong to a 1:1 cocrystal. Crystal suitable for single-crystal X-ray diffraction were selected directly from the sample as prepared.

### Refinement

H atoms bonded to N and O atoms were located in a difference map and allowed to ride on their parent atoms in the refinement cycles. Other H atoms were positioned geometrically and refined using a riding model.

### Crystal data

**Table:**

C_7_H_8_N_4_O_2_·C_7_H_6_O_4_	*Z* = 2
*M**_r_* = 334.29	*F*(000) = 348
Triclinic, *P*1	*D*_x_ = 1.567 Mg m^−^^3^
Hall symbol: -P 1	Melting point: 513 K
*a* = 7.0989 (14) Å	Mo *K*α radiation, λ = 0.71073 Å
*b* = 8.0543 (16) Å	Cell parameters from 2156 reflections
*c* = 13.034 (3) Å	θ = 2.6–31.0°
α = 86.08 (3)°	µ = 0.13 mm^−^^1^
β = 81.27 (3)°	*T* = 110 K
γ = 74.14 (3)°	Needle, yellow
*V* = 708.3 (3) Å^3^	0.24 × 0.22 × 0.13 mm

### Data collection

**Table:**

Rigaku Saturn CCD area-deterctor diffractometer	3478 independent reflections
Radiation source: fine-focus sealed tube	3302 reflections with *I* > 2σ(*I*)
graphite	*R*_int_ = 0.021
ω scans	θ_max_ = 28.3°, θ_min_ = 2.6°
Absorption correction: multi-scan (Blessing, 1995)	*h* = −9→7
*T*_min_ = 0.971, *T*_max_ = 0.984	*k* = −10→10
10245 measured reflections	*l* = −17→17

### Refinement

**Table:**

Refinement on *F*^2^	Primary atom site location: structure-invariant direct methods
Least-squares matrix: full	Secondary atom site location: difference Fourier map
*R*[*F*^2^ > 2σ(*F*^2^)] = 0.046	Hydrogen site location: inferred from neighbouring sites
*wR*(*F*^2^) = 0.127	H atoms treated by a mixture of independent and constrained refinement
*S* = 1.08	*w* = 1/[σ^2^(*F*_o_^2^) + (0.0749*P*)^2^ + 0.2093*P*] where *P* = (*F*_o_^2^ + 2*F*_c_^2^)/3
3478 reflections	(Δ/σ)_max_ < 0.001
235 parameters	Δρ_max_ = 0.36 e Å^−^^3^
0 restraints	Δρ_min_ = −0.33 e Å^−^^3^
0 constraints	

### Special details

**Table:**

Geometry. All e.s.d.'s (except the e.s.d. in the dihedral angle between two l.s. planes) are estimated using the full covariance matrix. The cell e.s.d.'s are taken into account individually in the estimation of e.s.d.'s in distances, angles and torsion angles; correlations between e.s.d.'s in cell parameters are only used when they are defined by crystal symmetry. An approximate (isotropic) treatment of cell e.s.d.'s is used for estimating e.s.d.'s involving l.s. planes.
Refinement. Refinement of *F*^2^ against ALL reflections. The weighted *R*-factor *wR* and goodness of fit *S* are based on *F*^2^, conventional *R*-factors *R* are based on *F*, with *F* set to zero for negative *F*^2^. The threshold expression of *F*^2^ > σ(*F*^2^) is used only for calculating *R*-factors(gt) *etc*. and is not relevant to the choice of reflections for refinement. *R*-factors based on *F*^2^ are statistically about twice as large as those based on *F*, and *R*- factors based on ALL data will be even larger.

### Fractional atomic coordinates and isotropic or equivalent isotropic displacement parameters (Å^2^)

**Table:**

	*x*	*y*	*z*	*U*_iso_*/*U*_eq_	
O5	0.17595 (15)	0.25838 (12)	0.08234 (7)	0.0256 (2)	
O3	0.01074 (15)	0.59680 (12)	0.09419 (7)	0.0271 (2)	
O4	0.00547 (15)	0.72663 (12)	0.24149 (7)	0.0271 (2)	
O6	0.30321 (16)	0.26347 (12)	0.48941 (7)	0.0294 (2)	
C8	0.14294 (17)	0.42610 (14)	0.23596 (9)	0.0181 (2)	
C13	0.17542 (18)	0.42529 (15)	0.33978 (9)	0.0199 (2)	
H13	0.1353	0.5278	0.3757	0.024*	
C14	0.04741 (18)	0.58978 (15)	0.18344 (9)	0.0202 (2)	
C10	0.29140 (18)	0.11806 (15)	0.23356 (10)	0.0217 (3)	
H10	0.3297	0.0145	0.1988	0.026*	
C11	0.32413 (19)	0.11938 (15)	0.33503 (10)	0.0220 (3)	
H11	0.3851	0.0169	0.3679	0.026*	
C9	0.20119 (18)	0.27073 (15)	0.18233 (9)	0.0195 (2)	
C12	0.26646 (19)	0.27340 (15)	0.38900 (9)	0.0213 (2)	
O2	0.28704 (13)	0.57656 (11)	0.56702 (7)	0.0221 (2)	
O1	0.50287 (14)	0.39024 (11)	0.87972 (7)	0.0244 (2)	
N3	0.23273 (15)	0.78018 (12)	0.68830 (8)	0.0181 (2)	
N4	0.39267 (15)	0.48739 (12)	0.72414 (8)	0.0187 (2)	
N2	0.19067 (16)	0.97032 (13)	0.83320 (8)	0.0207 (2)	
C1	0.41682 (17)	0.51168 (15)	0.82663 (9)	0.0186 (2)	
C2	0.30251 (17)	0.61332 (15)	0.65492 (9)	0.0176 (2)	
C5	0.33462 (17)	0.68618 (15)	0.85505 (9)	0.0183 (2)	
C3	0.24950 (17)	0.81358 (15)	0.78778 (9)	0.0177 (2)	
N1	0.32986 (16)	0.76825 (13)	0.94525 (8)	0.0206 (2)	
C6	0.1586 (2)	0.91951 (15)	0.61444 (9)	0.0232 (3)	
H6A	0.0502	1.0050	0.6495	0.035*	
H6B	0.1146	0.8733	0.5593	0.035*	
H6C	0.2626	0.9716	0.5862	0.035*	
C7	0.4852 (2)	0.31464 (15)	0.68265 (10)	0.0251 (3)	
H7A	0.3879	0.2508	0.6880	0.038*	
H7B	0.5893	0.2551	0.7217	0.038*	
H7C	0.5391	0.3250	0.6111	0.038*	
C4	0.24348 (19)	0.93614 (15)	0.92880 (9)	0.0225 (3)	
H4	0.2224	1.0198	0.9781	0.027*	
H5	0.111 (3)	0.366 (3)	0.0573 (16)	0.048 (6)*	
H6	0.289 (3)	0.372 (3)	0.5139 (16)	0.046 (5)*	
H4A	−0.063 (4)	0.834 (3)	0.2064 (19)	0.069 (7)*	
H1	0.383 (3)	0.714 (3)	1.0054 (15)	0.040 (5)*	

### Atomic displacement parameters (Å^2^)

**Table:**

	*U*^11^	*U*^22^	*U*^33^	*U*^12^	*U*^13^	*U*^23^
O5	0.0382 (5)	0.0190 (4)	0.0179 (4)	−0.0011 (4)	−0.0094 (4)	−0.0033 (3)
O3	0.0373 (5)	0.0208 (4)	0.0222 (4)	−0.0018 (4)	−0.0123 (4)	−0.0001 (3)
O4	0.0395 (5)	0.0152 (4)	0.0233 (4)	0.0025 (4)	−0.0105 (4)	−0.0035 (3)
O6	0.0512 (6)	0.0186 (4)	0.0205 (5)	−0.0070 (4)	−0.0162 (4)	0.0000 (3)
C8	0.0198 (5)	0.0149 (5)	0.0192 (5)	−0.0026 (4)	−0.0049 (4)	−0.0002 (4)
C13	0.0244 (6)	0.0155 (5)	0.0195 (5)	−0.0034 (4)	−0.0053 (4)	−0.0021 (4)
C14	0.0229 (5)	0.0157 (5)	0.0212 (5)	−0.0024 (4)	−0.0045 (4)	−0.0017 (4)
C10	0.0277 (6)	0.0142 (5)	0.0221 (6)	−0.0019 (4)	−0.0052 (5)	−0.0035 (4)
C11	0.0278 (6)	0.0157 (5)	0.0218 (6)	−0.0030 (4)	−0.0071 (5)	0.0006 (4)
C9	0.0222 (5)	0.0190 (5)	0.0171 (5)	−0.0037 (4)	−0.0047 (4)	−0.0028 (4)
C12	0.0285 (6)	0.0189 (5)	0.0181 (5)	−0.0066 (5)	−0.0073 (4)	−0.0006 (4)
O2	0.0307 (5)	0.0190 (4)	0.0158 (4)	−0.0032 (3)	−0.0069 (3)	−0.0023 (3)
O1	0.0324 (5)	0.0177 (4)	0.0206 (4)	−0.0001 (3)	−0.0089 (4)	0.0008 (3)
N3	0.0235 (5)	0.0142 (4)	0.0157 (5)	−0.0018 (4)	−0.0061 (4)	−0.0003 (3)
N4	0.0251 (5)	0.0134 (4)	0.0163 (5)	−0.0011 (4)	−0.0061 (4)	−0.0016 (4)
N2	0.0265 (5)	0.0158 (5)	0.0185 (5)	−0.0018 (4)	−0.0055 (4)	−0.0027 (4)
C1	0.0216 (5)	0.0174 (5)	0.0164 (5)	−0.0041 (4)	−0.0033 (4)	−0.0007 (4)
C2	0.0204 (5)	0.0156 (5)	0.0160 (5)	−0.0027 (4)	−0.0034 (4)	−0.0008 (4)
C5	0.0224 (5)	0.0168 (5)	0.0150 (5)	−0.0029 (4)	−0.0047 (4)	−0.0013 (4)
C3	0.0206 (5)	0.0160 (5)	0.0163 (5)	−0.0034 (4)	−0.0039 (4)	−0.0016 (4)
N1	0.0282 (5)	0.0170 (5)	0.0152 (5)	−0.0016 (4)	−0.0060 (4)	−0.0018 (4)
C6	0.0318 (6)	0.0166 (5)	0.0203 (5)	−0.0023 (5)	−0.0092 (5)	0.0020 (4)
C7	0.0359 (7)	0.0131 (5)	0.0236 (6)	0.0010 (5)	−0.0083 (5)	−0.0040 (4)
C4	0.0288 (6)	0.0171 (5)	0.0196 (6)	−0.0010 (4)	−0.0054 (5)	−0.0039 (4)

### Geometric parameters (Å, °)

**Table:**

O5—C9	1.3561 (14)	N3—C2	1.3754 (15)
O5—H5	0.92 (2)	N3—C6	1.4649 (15)
O3—C14	1.2245 (15)	N4—C2	1.3957 (15)
O4—C14	1.3199 (15)	N4—C1	1.4055 (14)
O4—H4A	0.99 (3)	N4—C7	1.4682 (15)
O6—C12	1.3658 (14)	N2—C4	1.3444 (15)
O6—H6	0.92 (2)	N2—C3	1.3629 (15)
C8—C9	1.4052 (16)	C1—C5	1.4179 (16)
C8—C13	1.4060 (16)	C5—C3	1.3733 (16)
C8—C14	1.4817 (17)	C5—N1	1.3792 (14)
C13—C12	1.3828 (17)	N1—C4	1.3408 (16)
C13—H13	0.9300	N1—H1	0.95 (2)
C10—C11	1.3783 (16)	C6—H6A	0.9600
C10—C9	1.3983 (17)	C6—H6B	0.9600
C10—H10	0.9300	C6—H6C	0.9600
C11—C12	1.3984 (17)	C7—H7A	0.9600
C11—H11	0.9300	C7—H7B	0.9600
O2—C2	1.2306 (14)	C7—H7C	0.9600
O1—C1	1.2321 (15)	C4—H4	0.9300
N3—C3	1.3710 (14)		
			
C9—O5—H5	109.4 (13)	O1—C1—N4	120.78 (11)
C14—O4—H4A	113.1 (14)	O1—C1—C5	127.75 (11)
C12—O6—H6	111.2 (13)	N4—C1—C5	111.46 (10)
C9—C8—C13	119.63 (11)	O2—C2—N3	121.22 (11)
C9—C8—C14	120.17 (11)	O2—C2—N4	121.22 (10)
C13—C8—C14	120.20 (11)	N3—C2—N4	117.56 (10)
C12—C13—C8	120.56 (11)	C3—C5—N1	105.65 (10)
C12—C13—H13	119.7	C3—C5—C1	122.98 (10)
C8—C13—H13	119.7	N1—C5—C1	131.26 (11)
O3—C14—O4	123.21 (11)	N2—C3—N3	126.79 (11)
O3—C14—C8	122.79 (11)	N2—C3—C5	110.99 (10)
O4—C14—C8	114.00 (10)	N3—C3—C5	122.21 (11)
C11—C10—C9	120.70 (11)	C4—N1—C5	106.66 (10)
C11—C10—H10	119.7	C4—N1—H1	128.1 (12)
C9—C10—H10	119.7	C5—N1—H1	125.2 (12)
C10—C11—C12	120.60 (11)	N3—C6—H6A	109.5
C10—C11—H11	119.7	N3—C6—H6B	109.5
C12—C11—H11	119.7	H6A—C6—H6B	109.5
O5—C9—C10	116.96 (10)	N3—C6—H6C	109.5
O5—C9—C8	123.97 (11)	H6A—C6—H6C	109.5
C10—C9—C8	119.07 (11)	H6B—C6—H6C	109.5
O6—C12—C13	123.61 (11)	N4—C7—H7A	109.5
O6—C12—C11	116.95 (11)	N4—C7—H7B	109.5
C13—C12—C11	119.44 (11)	H7A—C7—H7B	109.5
C3—N3—C2	118.91 (10)	N4—C7—H7C	109.5
C3—N3—C6	121.52 (10)	H7A—C7—H7C	109.5
C2—N3—C6	119.35 (10)	H7B—C7—H7C	109.5
C2—N4—C1	126.83 (10)	N1—C4—N2	112.63 (11)
C2—N4—C7	116.29 (10)	N1—C4—H4	123.7
C1—N4—C7	116.70 (10)	N2—C4—H4	123.7
C4—N2—C3	104.07 (10)		

### Hydrogen-bond geometry (Å, °)

**Table:**

*D*—H···*A*	*D*—H	H···*A*	*D*···*A*	*D*—H···*A*
N1—H1···O1^i^	0.95 (2)	1.85 (2)	2.8000 (16)	177.2 (17)
O6—H6···O2	0.92 (2)	1.83 (2)	2.7478 (14)	173.9 (18)
O5—H5···O3^ii^	0.92 (2)	2.24 (2)	2.8503 (16)	122.6 (17)
O5—H5···O3	0.92 (2)	1.87 (2)	2.6617 (15)	142.3 (19)
O4—H4A···N2^iii^	0.99 (3)	1.68 (3)	2.6596 (16)	171 (2)

Symmetry codes: (i) −*x*+1, −*y*+1, −*z*+2; (ii) −*x*, −*y*+1, −*z*; (iii) −*x*, −*y*+2, −*z*+1.
